# Types of skin afferent fibers and spinal opioid receptors that contribute to touch-induced inhibition of heart rate changes evoked by noxious cutaneous heat stimulation

**DOI:** 10.1186/s12990-015-0001-x

**Published:** 2015-02-12

**Authors:** Nobuhiro Watanabe, Mathieu Piché, Harumi Hotta

**Affiliations:** Department of Autonomic Neuroscience, Tokyo Metropolitan Institute of Gerontology, 35-2 Sakaecho, Itabashi-ku, Tokyo 173-0015 Japan; Department of Chiropractic, Université du Québec à Trois-Rivières, 3351 Boul. Des Forges, C.P 500, Trois-Rivières, Québec G9A 5H7 Canada

**Keywords:** Touch, Skin, Low-threshold mechanoreceptors, Noxious stimulation, Cardiovascular system, Spinal cord, μ-opioid receptor

## Abstract

**Background:**

In anesthetized rats and conscious humans, a gentle touch using a soft disc covered with microcones (with a texture similar to that of a finger), but not with a flat disc, inhibits nociceptive somatocardiac reflexes. Such an inhibitory effect is most reliably evoked when touch is applied to the skin ipsilateral and closest to nociceptive inputs. However, the mechanism of this inhibition is not completely elucidated. We aimed to clarify the types of cutaneous afferent fibers and spinal opioid receptors that contribute to antinociceptive effects of microcone touch.

**Results:**

The present study comprised two experiments with urethane-anesthetized rats. In the first experiment, unitary activity of skin afferent fibers was recorded from the saphenous nerve, and responses to a 10-min touch using a microcone disc and a flat disc (control) were compared. Greater discharge rate during microcone touch was observed in low-threshold mechanoreceptive Aδ and C afferent units, whereas many Aβ afferents responded similarly to the two types of touch. In the second experiment, the effect of an intrathecal injection of opioid receptor antagonists on the inhibitory effects of microcone touch on heart rate responses to noxious heat stimulation was examined. The magnitude of the heart rate response was significantly reduced by microcone touch in rats that received saline or naltrindole (δ-opioid receptor antagonist) injections. However, such an inhibition was not observed in rats that received naloxone (non-selective opioid receptor antagonist) or Phe-Cys-Tyr-Trp-Orn-Thr-Pen-Thr-NH_2_ (CTOP; μ-opioid receptor antagonist) injections.

**Conclusions:**

Microcone touch induced greater responses of low-threshold mechanoreceptive Aδ and C afferent units than control touch. The antinociceptive effect of microcone touch was abolished by intrathecal injection of μ-opioid receptor antagonist. These results suggest that excitation of low-threshold mechanoreceptive Aδ and C afferents produces the release of endogenous μ-opioid ligands in the spinal cord, resulting in the inhibition of nociceptive transmission that contributes to somatocardiac reflexes.

## Background

Noxious stimuli are transmitted to the cerebral cortex and perceived as “pain,” such information is integrated in the spinal cord and brain stem and influences autonomic nerve activity (somatoautonomic reflexes) [1-6]. Sympathetic nerve activity induced by noxious stimulation is thought to contribute to the onset and maintenance of some type of chronic pain [7]. Therefore, it is clinically important to manage autonomic responses to noxious stimuli.

Recently, we have found that somatoautonomic reflexes are inhibited by gently touching the skin in anesthetized rats [8,9] and conscious humans [10]. In these studies, the effect of touch using a soft disc filled with microcones, which array regularly with a constant pitch (a recently created device for continuously applying touch effect with a fingertip), was compared with that using a disc without microcones (i.e., a flat surface). In conscious humans, microcone touch applied to the inner ankle inhibited cardiovascular responses to noxious heat stimulation applied to the plantar foot, whereas flat disc touch had no influence [10]. Despite such different effects on the cardiovascular responses, texture discrimination was not possible [10]. A comparison of the human brain activity (glucose metabolism) during these two types of touch using positron emission tomography revealed no difference in the somatosensory cortex [11]. In anesthetized rats, the somatocardiac sympathetic reflex evoked by the activation of tibial C afferent fibers (the C-reflex) was selectively inhibited by microcone touch applied to the thigh [8,9]. This inhibitory effect on the C-reflex gradually appeared following the onset of continuous touch and slowly disappeared after termination of the touch. However, such an effect was not elicited by the touch using a flat disc [8].

Skin afferent fibers excited by microcone touch include low-threshold mechanoreceptive Aβ afferent units in the skin, which are important for tactile perception, as well as low-threshold mechanoreceptive Aδ and C afferent units [8]. Considering that the inhibitory effect on somatoautonomic reflexes was influenced by a slight difference in texture that does not affect sensation and metabolism in the somatosensory cortex, Aδ and C afferent units, but not Aβ afferent units, may contribute to the inhibitory effect of touch. Therefore, the present study first aimed to identify the type of cutaneous afferent units that contribute to the inhibition of somatoautonomic reflexes by comparing low-threshold mechanoreceptive Aβ, Aδ, and C afferent units responses to microcone touch with those to flat disc touch [8,10].

The inhibition by microcone touch appears to be a segmental inhibition via the spinal opioid system, because the effect of touch was most reliably evoked when touch was applied to ipsilateral and closer in location to the nociceptive input location and was attenuated by an intravenous administration of opioid receptor antagonist [8]. To date, a few studies have reported that intrathecal (i.t.) administration of exogenous opioids inhibits somatoautonomic reflexes [12-14]. For example, i.t. administration of μ- and δ-opioid receptor agonists inhibited cardiovascular responses to noxious heat stimulation in lightly anesthetized rats, but the κ-opioid receptor agonist did not [13]. Hence, the spinal μ- and/or δ-opioid receptors may contribute to the effect of touch. Therefore, the second aim of this study was to examine in deeply anesthetized rats, the different subtypes of spinal opioid receptors contributing to the effect of microcone touch on the cardiac response induced by noxious cutaneous heat stimulation. In the present study, we found that (1) microcone touch induced greater responses of low-threshold mechanoreceptive Aδ and C afferent units and (2) spinal μ-opioid receptors contributed to an inhibitory effect of microcone touch on heart rate (HR) response to noxious heat stimulation. A part of this study has been reported elsewhere as an abstract [15].

## Results

### Comparison of unitary activity between different types of touch

**Figure 1:**
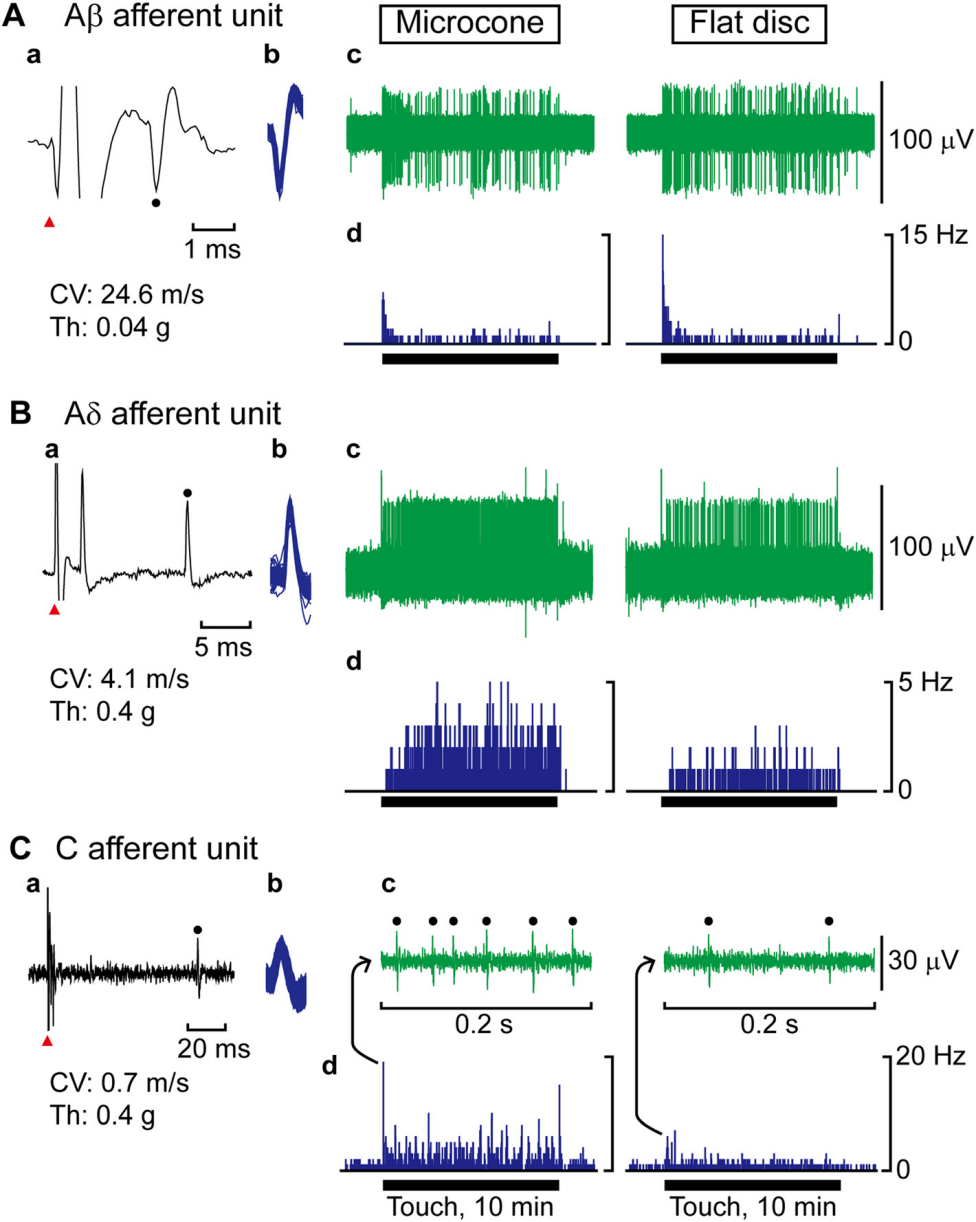
**Specimen recordings of unitary activity from the saphenous nerve.** Action potentials evoked by electrical stimulation applied on the receptive field (**A**a, **B**a, **C**a). A red triangle indicates the onset of electrical stimulation and a filled circle indicates a peak of recorded action potential. Conduction velocity (CV) was calculated based on a latency and an interelectrode distance. Mechanical threshold (Th) was measured by stimulating the receptive field of the recorded unit with von Frey filaments. Superimposed action potentials during a trial of microcone touch (**A**b, **B**b, **C**b). Recording of each afferent unit activity in responses to a 10-min touch using a microcone (left) and flat disc (right) (**A**c, **B**c). For C afferent unit activity, only an enlarged view at the onset of touch is presented and the action potentials of the unit are indicated by filled circles (**C**c). The histogram illustrates the discharge rate every second (**A**d, **B**d, **C**d). A horizontal line below each histogram indicates a period of touch (10 min).

To compare skin afferent unit responses to microcone touch with that to flat disc touch, unitary activity was recorded from the saphenous nerve in 17 anesthetized rats. In total, 40 low-threshold mechanoreceptive afferent units with slowly adapting properties were recorded. The conduction velocity that was obtained by applying electrical stimulation near the receptive field of recorded units was Aβ: 30.5 ± 1.5 m/s (13 units from 8 rats), Aδ: 10.7 ± 1.0 m/s (12 units from 7 rats), and C: 0.74 ± 0.03 m/s (15 units from 9 rats). In all measured units, the mechanical threshold obtained with von Frey filaments was ≤0.4 g (i.e., within the innocuous range), and the mean discharge rate during touch was <4 Hz. In Aβ and Aδ afferent units, some units exhibited a dynamic response at the onset of touch (SAI; 11 Aβ units as shown in Figure 1A, 7 Aδ units) and some did not (SAII; 2 Aβ units, 5 Aδ units as shown in Figure 1B). The characteristics of abovementioned recorded units are in accordance with those of our previous study [8].

Microcone touch or flat disc touch was applied to the receptive fields of recorded units for 10 min continuously. As shown in examples of recorded units of each group in Figure 1, responsiveness of Aβ afferent units hardly differed for the microcone and flat disc touch (Figure 1Ac,d), whereas Aδ and C afferent units exhibited greater responses to microcone touch (Figure 1Bc,d and 1Cc,d, respectively). There were no differences in response patterns (presence or absence of dynamic response) to either type of touch in any units.

**Figure 2:**
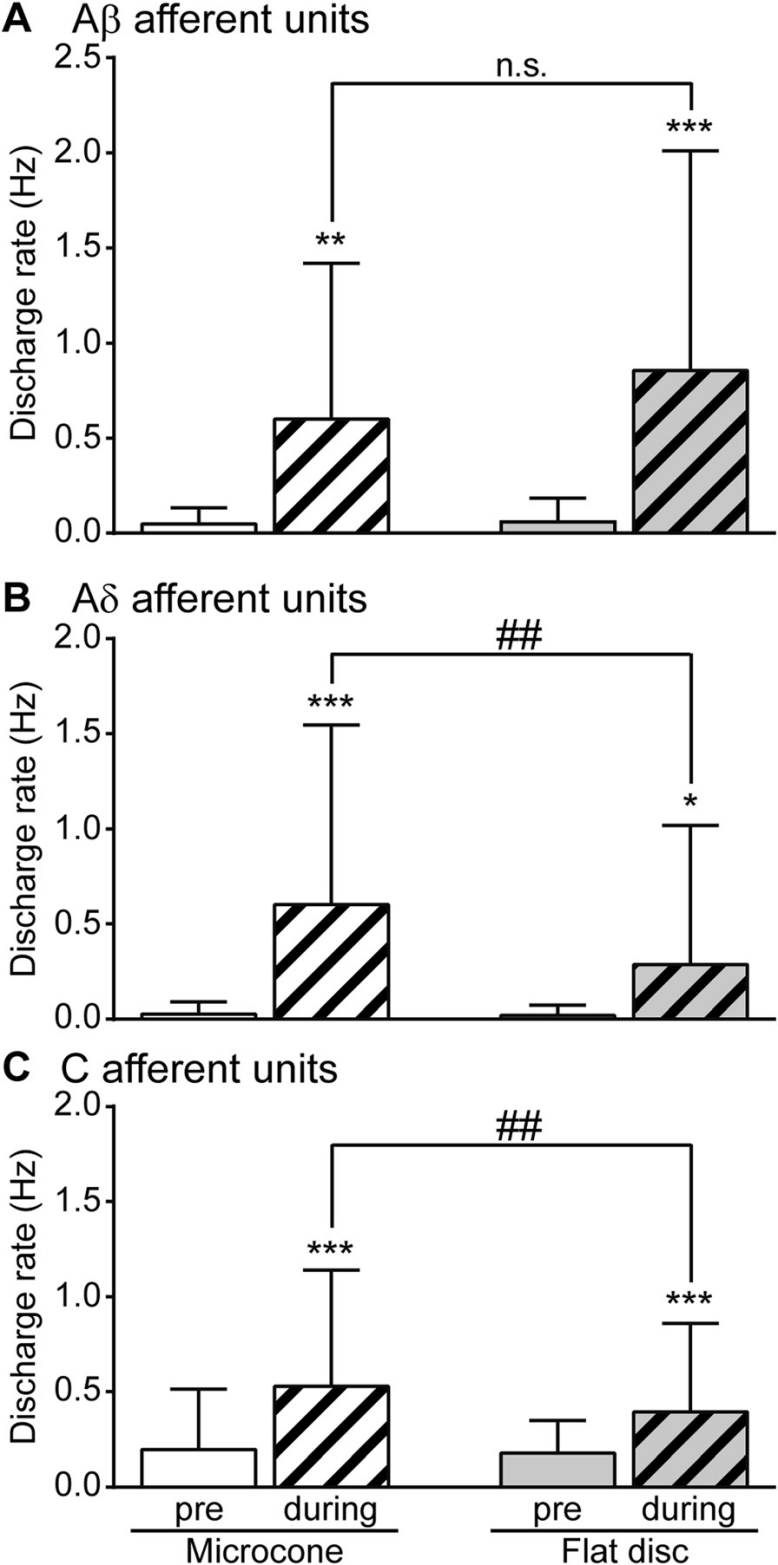
**Comparison of unitary activity of skin afferents in response to microcone and flat disc touch.** A hatched column indicates data obtained during touch. Data are expressed as the mean ± the standard deviation. The discharge rate of Aβ, Aδ, and C afferent units was compared (**A**-**C**, respectively) using a 2 × 2 repeated measures analysis of variance (ANOVA) with “conditions” (pre-touch and during touch) and “touch type” (microcone and flat disc) followed by the Bonferroni’s multiple comparison test. The results showed no significant interaction between “conditions” and “touch type” was observed in Aβ afferent units (*p* = 0.16); however, significant interactions were observed in Aδ and C afferent units (*p* = 0.032 and *p* = 0.048, respectively). * *p* < 0.05, ** *p* < 0.01, *** *p* < 0.001; a significant difference from a pre-touch value, ## *p* < 0.01; a significant difference between microcone and flat disc touch. n.s.; not significant.

Of 13 Aβ afferent units, only 4 units (including two SAII units) exhibited greater response to microcone touch, whereas the majority (7 units) showed no difference and 2 units exhibited smaller response to microcone touch. The discharge rate of Aβ afferent units during microcone touch and flat disc touch was 0.60 ± 0.82 Hz and 0.86 ± 1.20 Hz (the mean ± the standard deviation), respectively, and was not significantly different (*p* = 0.099) (Figure 2A). In contrast, in Aδ afferent units, of 12 units, most units (except one SAI unit and two SAII units) exhibited greater response to microcone touch. The discharge rate of Aδ afferent units during microcone touch was twice as high as that during flat disc touch (0.60 ± 0.94 Hz and 0.29 ± 0.73 Hz, respectively, *p* = 0.0093) (Figure 2B). Similarly, the discharge rate of C afferent units during microcone touch was greater than that during flat disc touch (0.53 ± 0.60 Hz and 0.39 ± 0.46 Hz, respectively, *p* = 0.0064) (Figure 2C).

### The contribution of spinal opioid receptors to the effect of microcone touch on noxious heat-induced HR response

#### Inhibition of noxious-heat induced HR response by microcone touch

**Figure 3:**
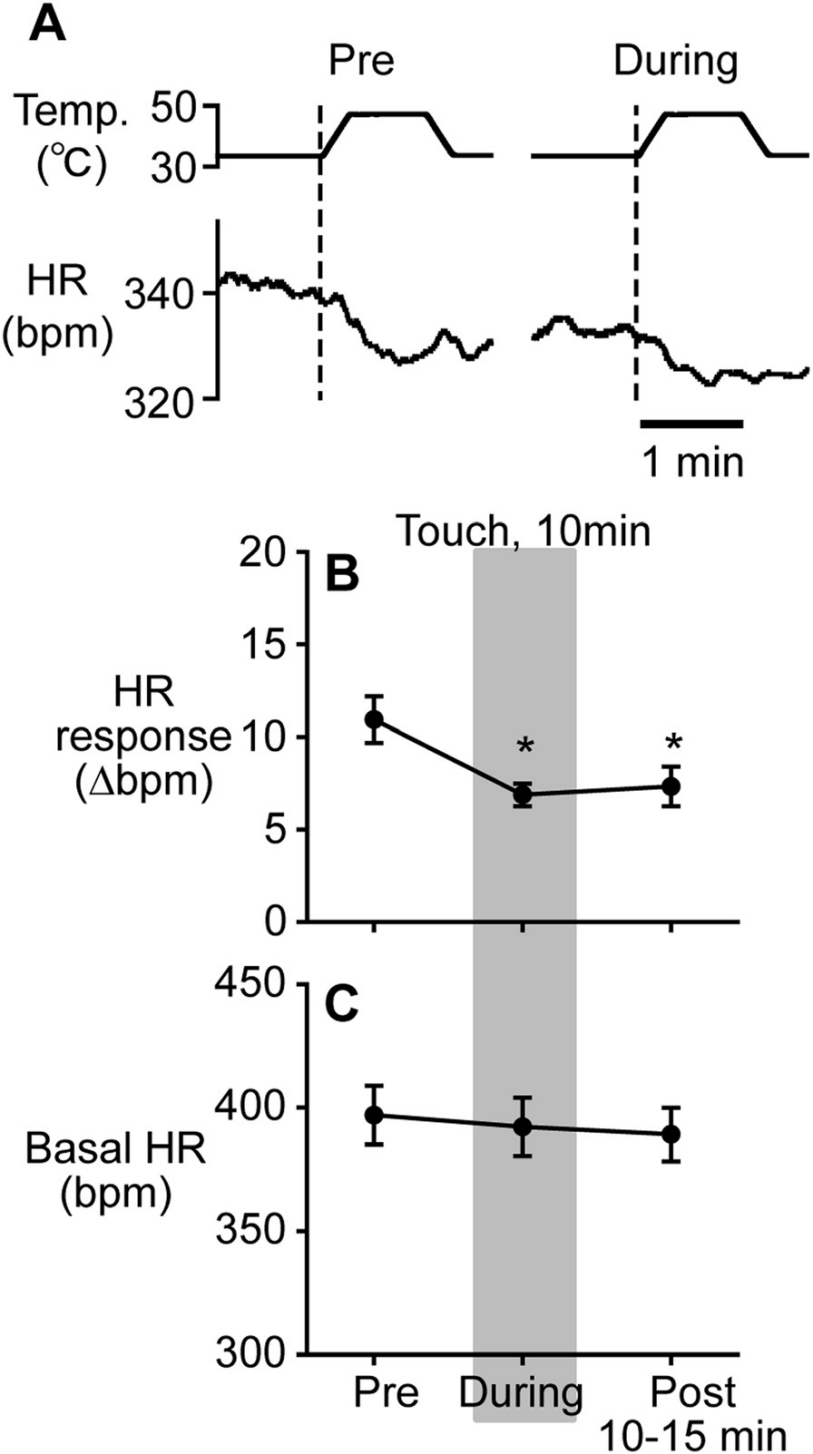
**The effect of microcone touch in saline-injected rats.** Specimen records of heart rate (HR) response to heat stimulation **(A)**, and averages of changes in HR response **(B)** and in basal HR **(C)** before, during, and after touch. Statistical analysis was performed using the one-way repeated measures ANOVA followed by the Bonferroni’s multiple comparison test for HR response. The analysis showed that there was a significant main effect of “conditions” (pre-, during, and post-touch) (*p* = 0.013). * *p* < 0.05; a significant difference from a pre-touch value. Data are expressed as the mean ± the standard error of the mean.

To evaluate the inhibitory effect of microcone touch on nociceptive transmission, we examined the effect of touch on HR response induced by noxious heat stimulation. Noxious heat stimulation applied to the lower back and rumps of the anesthetized rat altered HR (Figure 3A). In rats i.t.-injected with saline (n = 7), HR responses to heat stimulation were recorded twice without touch in each rat and a total of 14 recordings was obtained. HR increased in 3 recordings for the first stimulation, but HR decreased in the other 11 recordings. The magnitude of change with respect to basal HR (average HR of 1 min before the start of heat stimulation) was 5–17.5 bpm. In response to heat stimulation, blood pressure was altered in the same direction as HR changed.

In rats i.t.-injected with saline, 10 min of microcone touch applied unilaterally to the right inner thigh reduced the magnitude of HR response to noxious heat stimulation (Figure 3A). The result of saline-injected rats (n = 7) is summarized in Figure 3B. The heat-induced HR response (Δbpm) was significantly inhibited during microcone touch by approximately 37% (from 10.9 ± 1.2 bpm to 6.9 ± 0.6 bpm, *p* = 0.013). Such an inhibition continued even at 10–15 min after the touch was terminated (7.3 ± 1.1 bpm, *p* = 0.026).

The effect of flat disc touch on heat-induced HR response was also tested in 4 rats without the spinal catheter. As observed in our previous studies [8,10], HR response (9.5 ± 1.6 bpm) was not significantly influenced during flat disc touch (8.3 ± 1.8 bpm, *p* = 0.80) or after touch (9.0 ± 1.6 bpm, *p* > 0.99).

#### Contribution of spinal opioid receptors to an inhibitory effect of touch

To examine a possible contribution of the endogenous opioid system in the spinal cord to an inhibitory effect of microcone touch on noxious heat-induced HR response, opioid receptor antagonists were administered i.t. in advance. In the groups injected with naloxone (non-selective opioid receptor antagonist), Phe-Cys-Tyr-Trp-Orn-Thr-Pen-Thr-NH_2_ (CTOP; μ-opioid receptor antagonist), and naltrindole (δ-opioid receptor antagonist), the average of HR responses (Δbpm) before microcone touch were 10.6 ± 2.0, 10.3 ± 2.4, and 10.5 ± 2.1 bpm, respectively. The magnitude of HR response before touch did not differ between groups (including the saline-injected group).

**Figure 4:**
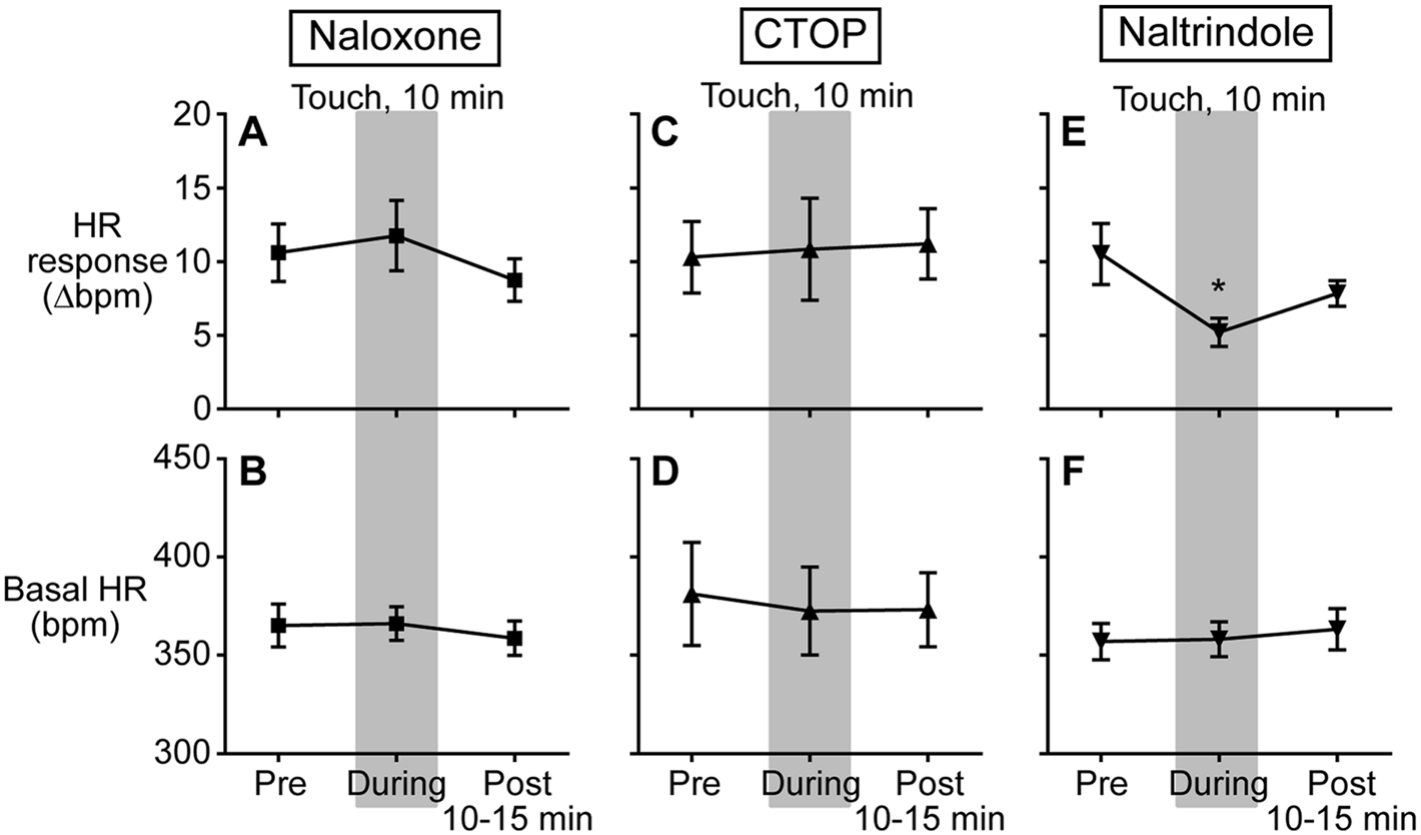
**Influence of opioid receptor antagonists on the effect of microcone touch.** Averages of changes in heart rate (HR) response to heat stimulation **(A, C, E)** and basal HR **(B, D, F)** before, during, and after touch in rats injected with naloxone, CTOP, and naltrindole, respectively. The influence of i.t.-injected drugs on the effect of touch on heat-induced HR response and basal HR was examined using a 3 × 4 mixed model ANOVA followed by the Bonferroni’s multiple comparison test; “conditions” (pre-touch, during touch, and post-touch) as the within-subject factor and “drugs” (saline, naloxone, CTOP, and naltrindole) as the between-subject factor. The analysis shows that a significant interaction was observed in HR response (*p* = 0.02), but not basal HR (*p* = 0.20). See Figure 3 for further details.

In naloxone-injected rats (n = 5), the HR response was 10.6 ± 2.0 bpm (before touch), 11.8 ± 2.4 bpm (during touch), and 8.8 ± 1.4 bpm (after touch), and it was not significantly influenced by microcone touch (Figure 4A). In CTOP-injected rats (n = 5), HR response was not inhibited by touch (Figure 4C). In contrast, in naltrindole-injected rats (n = 5), HR response (10.5 ± 2.1 bpm before touch) was significantly inhibited during touch (5.2 ± 1.0 bpm, *p* = 0.003) and tended to be attenuated following the cessation of touch (7.9 ± 0.9 bpm, *p* = 0.18) (Figure 4E). The magnitude of HR response inhibition by touch in the naltrindole-injected group was comparable to the saline group (Figure 3B).

#### The effect of microcone touch on basal HR

To examine a potential influence of microcone touch on basal HR, basal HRs obtained before, during, and after touch were compared. Basal HR before touch was 316–470 bpm. Basal HR was not influenced by touch in any groups (saline, naloxone, CTOP, or naltrindole) and was not different among groups under any conditions (pre-touch, during, and post-touch) (Figures 3C and 4B, D, F).

Additionally, to examine an influence of i.t. administration on basal HR, basal HR recorded immediately before i.t. administration and before heat stimulation (i.e., after administration) was compared. Basal HR was not different before and after i.t. administration in any groups; saline (394.0 ± 12.0 and 380.3 ± 13.5 bpm, respectively, *p* = 0.23), naloxone (364.5 ± 11.3 and 374.0 ± 14.5 bpm, respectively, *p* = 0.34), CTOP (383.2 ± 28.4 and 365.1 ± 22.6 bpm, respectively, *p* = 0.18), or naltrindole (356.3 ± 8.3 and 366.3 ± 10.7 bpm, respectively, *p* = 0.33).

## Discussion

In the present study, we aimed to identify cutaneous afferent units that contribute to the effect of gentle mechanical cutaneous stimulation (touch) with microcones on somatocardiac reflexes. Furthermore, we investigated neural mechanisms related to the spinal opioid system for the effect of microcone touch on noxious heat-induced HR response. Two novel findings were obtained. First, among low-threshold mechanoreceptive afferent units with slowly adapting properties, the discharge rate of the Aδ and C afferent units induced by microcone touch was greater than that induced by control stimulation (flat disc). Second, the inhibitory effect of microcone touch on noxious heat-induced HR response was abolished by i.t. injection of a non-selective opioid receptor antagonist and a selective μ-opioid receptor antagonist, but not influenced by a selective δ-opioid receptor antagonist. Therefore, the present study results suggest that microcone touch-induced excitation of low-threshold cutaneous mechanoreceptive Aδ and C afferent units inhibited nociceptive transmission into autonomic reflex pathways via the spinal μ-opioid system.

### Afferent mechanisms for the effect of microcone touch

In the present study, the response of cutaneous afferent units to microcone touch was compared with that elicited by flat disc touch, which did not influence the somatocardiac reflexes previously reported [8,10] and confirmed in this study. As expected, such a comparison revealed that microcone touch induced a greater response of Aδ and C afferent units, but not of Aβ afferents.

Aβ afferent inputs ascend to the somatosensory cortex and are considered to be important for perception and discrimination of touch [16]. Thus, a similar discharge rate of Aβ afferents during the two types of touch is consistent with that of our previous studies with conscious humans showing that (1) glucose metabolism during touch using microcone and flat discs was not different in the somatosensory cortex [11], and (2) the types of touch applied were not perceptually distinguishable [10]. Furthermore, studies using electrical stimulation showed that nociceptive transmission is inhibited by high-frequency stimulation (5 Hz≤) of Aβ afferents [17,18], but not by low-frequency stimulation (≤0.5 Hz) [19]. Therefore, it is assumed that low-frequency activation of Aβ afferents induced by touch (microcone: 0.6 Hz, flat disc: 0.86 Hz) in this study does not elicit an inhibition of nociceptive transmission.

Low-threshold mechanoreceptive Aδ and C afferent units in hairy skin are sensitive to gentle mechanical stimuli, such as a breeze over the skin [16]. All experiments of the present study were performed in vivo using anesthetized rats. Therefore, body movement induced by pulsation and respiration may vibrate microcones on the skin [8] and elicit continuous stimulation where microcone touch was applied. Vallbo et al. [20] reported that some recorded low-threshold mechanoreceptive C afferent units stopped firing immediately (within 4 s) after the onset of stimulation using a probe with a rounded tip, and those units responded longer to touch with a finger. The texture of microcone and flat disc tools in the present study is presumably similar to that of finger skin and the stimulation probe in the study by Vallbo et al. [20], respectively. Therefore, it is speculated that a slight difference in texture, despite not being perceptually distinguishable, elicited different responses in the low-threshold mechanoreceptive Aδ and C afferent units.

There are a number of studies suggesting that excitation of Aδ and C afferents induced by electrical stimulation is related to an inhibition of nociceptive transmission in the spinal cord [19,21-25]. Ikeda et al. [19] reported that neuronal responses in the spinal dorsal horn to C afferent nerve stimulation are inhibited by excitation of Aδ and C afferents. In particular, such an inhibition is more robustly induced by afferent stimulation at 0.5 Hz than that at 0.2 Hz. Electrical stimulation at similar intensities may activate nerve fibers conveying temperature and nociceptive information. However, the slight difference in stimulation frequency producing a different inhibitory effect in that study [19] is similar to the difference in discharge rate during microcone touch and flat disc touch in the present study (Aδ: 0.6 Hz vs. 0.29 Hz, respectively, C: 0.53 Hz vs. 0.39 Hz) (Figure 2B, C). Therefore, the present study is the first showing the possibility that low-threshold mechanoreceptive Aδ and C afferent units contribute to the inhibition of nociceptive transmission.

### Touch-induced inhibition of somatoautonomic reflexes

In the present study, touch with microcone inhibited heat-induced HR response whereas touch with flat disc did not significantly influence HR response. This result is consistent with our previous studies [8,10] and the time course of microcone touch-induced inhibition on the heat-induced HR response was similar to that on the C-reflex in our previous study [8]. Although noxious heat stimulation primarily induced bradycardiac responses, the inhibitory (i.e., suppresive) effect of touch on cardiac responses observed in the present study was analogous to excitatory reflex discharges in anesthetized rats [8,9] and to tachycardiac (bradycardiac, in some cases) responses in conscious humans [10]. In addition, although the skin area where noxious stimulation was applied was different from our previous study (plantar foot [10] vs. lower back and rumps in the present study), an inhibitory effect was induced by touch applied to dermatomes close to noxious stimuli [26]. These findings suggest that the inhibitory effect of touch is a ubiquitous phenomenon that segmentally influences an ascending pathway of nociceptive inputs.

Somatocardiac reflexes are influenced by the location of noxious stimulation and the depth of anesthesia [1]. Noxious stimulation applied to more distal part of the body induces tachycardiac and pressor responses whereas stimulation to more proximal part of the body may cause bradycardiac and depressor responses [27,28]. In addition, according to Gibbs et al. [27], the frequency of bradycardiac and depressor response increases when the concentration of inhalation anesthetics becomes higher. Thus, it was assumed that bradycardiac responses were more likely to be caused due to experimental conditions in the present study (noxious heat stimulation applied to the lower back and rumps and deeper anesthesia than conventional depth [8,9]). A difference in cardiovascular responses to noxious stimulation (bradycardia or tachycardia) was assumed to be centrally mediated [27]. Since touch with microcone inhibits nociceptive transmission at the spinal level, both bradycardiac and tachycardiac responses to heat stimulation may be inhibited by the same mechanism.

The cardiac response induced by noxious heat stimulation applied to the lower back and rumps in the present study may have involved both spinal and supraspinal reflexes [29]. Among these reflex components, the supraspinal C-reflex plays a major role in HR responses [30]. The temperature of heat stimulation (46°C), which is similar to that in the present study, predominantly stimulated unmyelinated C afferent fibers [31]. Thus, our results suggest that microcone touch suppressed C afferent transmission triggered by noxious heat stimulation, which induced the supraspinal C-reflex, and consequently inhibited HR responses.

### The subtype of opioid receptor involved in the inhibitory effect of touch

The present study further found that i.t. administration of a selective μ-opioid receptor antagonist completely blocked the touch effect. This result clearly showed that activation of μ-opioid receptors in the spinal cord is responsible for the inhibitory effect of touch on somatocardiac reflexes. Our result is supported by previous studies demonstrating that i.t. administration of μ-opioid agonists inhibited the somatocardiac reflexes [13,14].

Although a contribution of spinal δ-opioid receptors to the touch effect was expected in the present study, i.t. administration of naltrindole (a δ-opioid receptor antagonist) did not influence the inhibitory effect of touch. This result suggests that spinal δ-opioid receptors do not contribute to the touch effect. A dose of 30 μg of naltrindole completely blocks the maximal effect of a δ-opioid receptor agonist on nociceptive behaviors [32], suggesting that the dose of naltrindole used in the present study (25 μg) sufficiently blocked spinal δ-opioid receptors. The previous studies showed that spinal δ-opioid agonists were also effective for inhibiting the somatocardiac reflexes [13,14]. However, the present study is supported by a previous study [14] stating that the effect of a δ-opioid agonist resulted from non-specific or cross-reactive binding to μ-opioid receptors, because only larger doses of δ-opioid agonist were effective (1000 times that of μ-opioid agonist). More recently, it has been found that δ-opioid receptors rarely exist in unmyelinated C fibers with transient receptor potential vanilloid 1 (TRPV1) channel [33,34], which are heat-sensitive nerve fibers. However, one study reported that δ-opioid receptors are present in many heat-sensitive neurons [35].

### Hypothesis on inhibitory pathway associated with the spinal μ-opioid system

**Figure 5:**
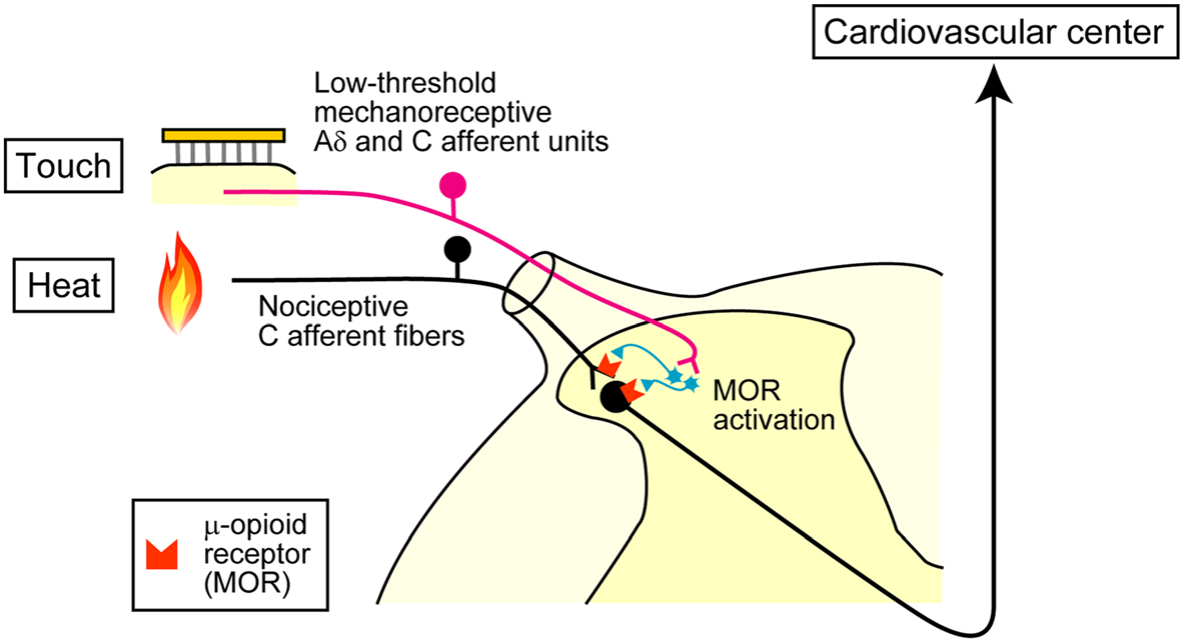
**A hypothesized mechanism of touch-induced inhibitory effect on spinal nociceptive transmission into somatocardiac reflex pathways.** Noxious heat-evoked sensory inputs were conveyed by nociceptive C afferent fibers and transmitted to the secondary neurons in the spinal cord. Low-threshold mechanoreceptive Aδ and C afferent units were excited by touch. Touch-induced sensory afferent excitation enhances the release of endogenous opioids from opioid-containing interneurons (in light blue) and/or primary afferents (not shown) in the superficial layers of the dorsal horn. Subsequently, μ-opioid receptors (MORs) are activated, resulting in an inhibition of nociceptive transmission into somatocardiac reflex pathways.

On the basis of the present results, we hypothesized that afferent inputs arising from low-threshold mechanoreceptive Aδ and C afferent units in hairy skin drive the spinal μ-opioid system (Figure 5). In particular, such an effect potentially occurs in the superficial layers (laminae I–II) of the spinal cord due to the following reasons: (1) the nociceptive Aδ and C afferent fiber terminals [36], (2) the low-threshold mechanoreceptive Aδ and C afferent unit terminals [16,36,37], and (3) high density of μ-opioid receptors [38,39] (primarily on the afferent terminals [40]) located in the superficial dorsal horn. It has been reported that μ-opioid receptors densly exist in TRPV1-positive unmyelinated afferents [34]. In addition, endogenous ligands for μ-opioid receptors (e.g., endomorphin-2 and enkephalins) are localized in the primary afferent terminals [41-44] and enkephalin-containing neurons are found in the superficial dorsal horn [45,46]. Thus, it may be possible that low-threshold mechanoreceptive Aδ and C afferent units produce the release of endogenous opioids. In the dorsal horn, a highly complex neural network is constituted by functionally and neurochemically distinct types of neurons [36,44,47-49]. Future studies will provide more detailed association of low-threshold mechanoreceptive Aδ and C afferent units with opioidergic neurons in the spinal cord.

### Clinical implications

Yaksh and Elide [50] demonstrated that in anesthetized cats, the release of methionine–enkephalin is increased by the excitation of Aδ and C afferents in the sciatic nerve using electrical stimulation. Our results suggest that activation of low-threshold mechanoreceptive Aδ and C afferents by touching the skin with microcones (and possibly with fingers) may enhance the release of endogenous opioids. Thus, our results may provide a neurological explanation for the clinical observation that microcone treatment is helpful in chronic pain relief in patients with fibromyalgia (personal communication with Katsutaro Nagata), accompanying with autonomic dysfunctions.

## Conclusions

In conclusion, activation of low-threshold mechanoreceptive Aδ and C afferent units in the hairy skin, induced by gentle mechanical cutaneous stimulation (touch) using microcones, may inhibit nociceptive transmission into autonomic reflex pathways via spinal μ-opioid receptors. These findings may help elucidating the role of cutaneous receptors in functions other than discriminative touch.

## Materials and methods

The experiments were performed using 43 adult male Wistar rats (285–455 g) bred at the Tokyo Metropolitan Institute of Gerontology (Tokyo, Japan). Experimental protocols were approved by the Animal Care and Use Committee of Tokyo Metropolitan Institute of Gerontology. The present study comprised two experiments described below. Both experiments were performed under urethane anesthesia. The initial induction was performed subcutaneously or intraperitoneally. Sufficiently deep anesthesia is indicated by the absence of whisker movement, the lack of corneal reflex, and stable blood pressure. Additional anesthetic (4%–19% of the initial dose) was given subcutaneously, intraperitoneally, or intravenously as required to maintain deep anesthesia throughout the experiment.

The preparatory surgery was described previously [8,9]. In brief, the carotid artery and jugular vein were catheterized for continuous arterial blood pressure recording and administration of anesthetics and supplemental fluids, respectively. The trachea was cannulated for artificial ventilation (SN-480-7; Shinano Seisakusho, Tokyo, Japan). Ventilation was controlled to maintain the end-tidal CO_2_ level at approximately 3% (Microcap; Oridion Medical, Jerusalem, Israel). Rectal temperature was maintained between 37.0°C and 37.5°C using a heat pad and lamp (ATB-1100; Nihon Kohden, Tokyo, Japan).

### Touch

**Figure 6:**
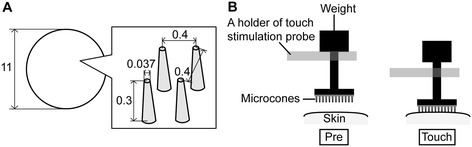
**A specification of microcone tool and a method for application of touch on the skin.** A specification of microcones is shown in an enlarged view of panel **A**. Approximately 400 microcones are arrayed on a circular disc, 11 mm diameter; the space between microcones is 0.4 mm. An application method for touch in panel **B** was referred to in our previous study [8].

The fur on the inner thigh, where touch was applied, was trimmed with a conventional clipper. Touch was applied using a soft elastomer disc containing approximately 400 microcones on a circular surface of 11-mm diameter (microcone touch) (Somareson I, Toyoresin, Shizuoka, Japan) (Figure 6A) or using a disc with a flat surface, composed of the same material (prepared by Toyoresin), as a control. The disc was applied with a 12 g weight for 10 min [8].

Touch stimulation was applied by placing an elastomer disc (microcone or flat disc), stuck on the tip of the touch stimulation probe, on the skin [8]. The touch stimulation probe was suspended on the holder with a weight attached on the top end of the probe (Figure 6B, left). Touch stimulation was applied by lowering the holder and the constant force was continuously applied as long as the weight remained detached from the holder (Figure 6B, right). At the onset and offset of touch stimulation, the holder was moved either using a robotic device or manually. In the case of manual operation, all stimuli were performed by the same experimenter and movement velocity was carefully controlled.

### Recording of unitary activity from cutaneous afferents

Unitary activity of cutaneous afferents was recorded from the saphenous nerve, and the responses to two types of touch (microcone and flat disc) were compared in 17 rats. Rats were anesthetized using urethane (1.1 g/kg, intraperitoneally). Gallamine triethiodide (20 mg/kg) was intravenously injected for immobilization during recordings.

Recording methods were described previously [8]. The thigh branch of the saphenous nerve was cut close to the inguinal ligament and covered with warm paraffin oil. The distal cut end of the nerve was placed on a bipolar platinum–iridium wire electrode for activity recording. The nerve was dissected using forceps until clear unitary activity (single or multiple units) was obtained from the individual dissected nerve. The obtained unitary activity was amplified 1,000-10,000 times (MEG-6100; Nihon Kohden) and then monitored visually (on an oscilloscope) and auditorily (through loudspeakers). The amplified signal was sampled at 20 kHz (Micro 1401 mkII; Cambridge Electronic Design, Cambridge, England) and stored on a personal computer for offline analyses, including spike sorting using Spike2 software (Cambridge Electronic Design).

In the present study, we focused on slowly adapting units with low-mechanical thresholds (≤0.4 g; i.e., within the innocuous range) responding to microcone touch, according to our previous result [8]. For this experiment, basically, mechanical threshold of the recorded afferent unit was first measured, responses of the unit to touch were then obtained, and the response to electrical stimulation was finally recorded to determine conduction velocity. The mechanical threshold of each unit was obtained using von Frey filaments (0.003–0.4 g). Unitary activity was recorded for 2 min before touch and for 10 min during touch. The location of touch was adjusted as the receptive field of recorded units is within touch area (11-mm diameter). The two types of touch were applied sequentially to the same skin area and the order of stimuli was randomized. Conduction velocity was calculated by recording the latency of electrically evoked action potentials and by measuring the inter-electrode distance after the end of the experiment. For the evoked action potential recording, the skin near the receptive field of the recorded units was stimulated electrically (square pulse, 0.5-ms duration) using a pair of needle electrodes. The identity of the action potentials evoked by touch and electrical stimulation was verified on the basis of the amplitude and shape of the recorded signals (e.g., Figure 1Aa,b). Classification of afferent groups was determined by the conduction velocity: Aβ was >15.6 m/s, Aδ was ≤15.6 m/s, and C was ≤2 m/s [8]. Responsiveness of low-threshold mechanoreceptive C afferent units may be reduced by repetitive stimulation with short rest periods (fatigue) [51,52]. In the present study, touch stimuli were separated by at least 5 min and we observed no systematic reduction in responsiveness of the afferent units.

### Noxious heat-induced HR response

To examine the effect of touch on noxious heat-induced HR response and the contribution of spinal opioid receptors to the effect of touch, 26 rats (5–6 months old) were used. Urethane (1.4 g/kg) was injected subcutaneously between the two scapulae, following an initial inhalation of 3% halothane for approximately 3 min. Preparatory surgery began at least 40 min following the initial urethane dose.

#### Heat stimulation

**Figure 7:**
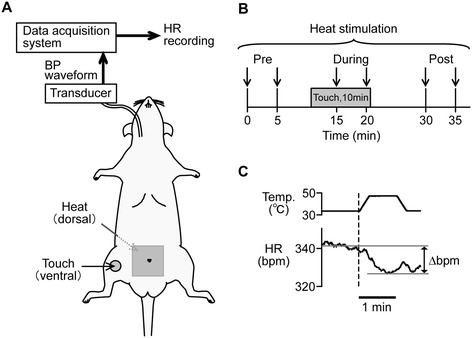
**Summary of experimental methods for examining the effect of touch on noxious heat-induced heart rate (HR) response.** Heat stimulation was applied to the center of the lower back and rumps and touch was applied to the right inner thigh **(A)**. A time point of heat stimulation is indicated by a down arrow **(B)**. HR response was determined as a maximal change of HR during heat stimulation with respect to basal HR (average HR of 1 min before the onset of heat stimulation) **(C)**.

Noxious heat stimulation was applied using a heat-stimulation device equipped with a Peltier thermode (DPS-777PH; Physio-Tech, Tokyo, Japan) [10]. The thermode (heat area: 3 cm × 3 cm) was placed under the center of the lower back and rumps of the rat where the skin had been gently trimmed (Figure 7A). Heat stimulation was applied twice for each of three conditions, pre-touch (at 0 and 5 min), during (at 15 and 20 min), and post-touch (at 30 and 35 min) (Figure 7B). The temperature of the thermode was maintained at 33°C during experiment. When heat stimulation was applied for 1 min, the temperature was increased at a rate of 1°C/sec to peak temperature (46–48°C). Before the application of touch, peak temperature was determined as the temperature that induced HR changes ranging between 5 and 20 bpm (cf. [29,53,54]), with respect to basal HR (provided below). The heat stimulation peak temperature was first set at 46°C and increased when necessary. The same peak temperature determined for each animal was used through the course of the experiment.

#### Determination of HR response

The instantaneous HR was calculated from recorded blood pressure waveforms (Figure 7A) (Spike2; Cambridge Electronic Design). The HR waveform was smoothed with a time constant of 5 s. The quantification of HR response was based on our previous study [10]. A HR response (Δbpm) was determined by the peak of HR changes (i.e., minimal or maximal value) during the peak temperature of the heat stimulation with respect to basal HR value, which was an average of HR recorded 1 min before heat stimulation (Figure 7C).

#### Blockage of spinal opioid receptors

The contribution of spinal opioid receptors to the effect of microcone touch was examined by i.t. administration of opioid receptor antagonists. Naloxone hydrochloride (non-selective-opioid receptor antagonist; 10 μg in 10 μL saline), Phe-Cys-Tyr-Trp-Orn-Thr-Pen-Thr-NH_2_ (CTOP, μ-opioid receptor antagonist; 240 ng in 10 μL saline), naltrindole hydrochloride (δ-opioid receptor antagonist; 25 μg in 10 μL saline) and saline (control) were used (all antagonists were purchased from Sigma-Aldrich, St. Louis, MO, USA). Doses of antagonists used in the present study sufficiently antagonize the effects of opioid receptor agonists [32,55,56]. The substances (10 μL) were administered through a catheter implanted in the subarachnoid space at the lumbar spinal cord level (SP-8; Natsume Seisakusho, Tokyo, Japan). The method for catheter implantation was based on previous studies [14,55,57]. The tip of the catheter was inserted through the atlanto-occipital membrane and was then positioned approximately 9 cm caudally (at the lumbar enlargement of the spinal cord). Catheter implantation was performed on the day of experiment. After each experiment, a laminectomy confirmed that the catheter did not penetrate the spinal cord. To avoid possible catheter blockage by blood clots, the tip of the catheter was filled with 2 μL of heparin (200 U/mL). Administration was performed manually at a rate of approximately 1 μL/10 s, guided by an auditory cue. Substance administration was completed at least 20 min before the first heat stimulation.

### Data analyses and statistics

#### Recording of unitary activity of cutaneous afferents

The discharge rate (Hz) of the afferent units was averaged for 2 min before touch and for 10 min during touch. The discharge rate was compared using a 2 × 2 repeated measures analysis of variance (ANOVA) with “conditions” (pre-touch and during touch) and “touch type” (microcone and flat disc) (Prism6; GraphPad Software Inc., La Jolla, CA, USA). Post hoc analysis was performed by the Bonferroni’s multiple comparison test. The statistical significance level was set at 5%. Data were expressed as the mean ± the standard error of the mean, unless otherwise stated.

#### Noxious heat-induced HR response

The values of HR response (Δbpm) and basal HR were averaged across the two stimuli for each condition (pre-touch, during touch, and post-touch). First, in saline-injected rats, the effect of microcone touch on noxious heat-induced HR responses was examined with a one-way repeated measures ANOVA, followed by the Bonferroni’s post-hoc comparisons test (Prism6). The effect of flat disc touch was also tested in the same way. The influence of i.t. administered drugs on the effect of touch on heat-induced HR response and basal HR was then examined using a 3 × 4 mixed model ANOVA with “conditions” (pre-touch, during touch, and post-touch) as the within-subject factor and “drugs” (saline, naloxone, CTOP, and naltrindole) as the between-subject factor (Prism6). Post hoc analysis was performed by the Bonferroni’s multiple comparison test. The influence of i.t. administration on basal HR was examined by comparing averages of HR recorded 1 min before i.t. administration and heat stimulation (after i.t. administration) using paired t-test. The statistical significance level was set at 5%. Data were expressed as the mean ± the standard error of the mean, unless otherwise stated.

## Abbreviations

ANOVA: Analysis of variance
HR: Heart rate
i.t.: intrathecal (intrathecally)
SAI: Slowly adapting unit with dynamic response at the onset of stimulation
SAII: Slowly adapting unit without dynamic response at the onset of stimulation
TRPV1: Transient receptor potential vanilloid 1
